# Experimental Method of Machining Gears with an Involute Profile Using CNC Lathe with Driven Tools

**DOI:** 10.3390/ma15031077

**Published:** 2022-01-29

**Authors:** Rafał Gołębski

**Affiliations:** Department Technology and Automation, Czestochowa University of Technology, Al. Armii Krajowej 21, 42-200 Częstochowa, Poland; rafal.golebski@pcz.pl

**Keywords:** CNC machining, driven tools, involute, lathe, outline, roughness, spur gear, surface integrity parameters

## Abstract

There are many ways of machining gears; the world’s manufacturers of machine tools have patented many methods that allow for the production of gears in an accurate and efficient way. In general, the patented methods require the use of kinematically complex and expensive CNC machine tools. These tools, moreover, the production of the technology itself, including the machining code, require the use of dedicated software. Therefore, it seems justified to strive for the application of kinematically simple and relatively cheap machines in the machining processes so as to increase the universality and availability of new machining methods. This paper presents a method of machining a spur gear with straight teeth with an involute profile using a basic CNC lathe DMG MORI CLX350V4 equipped with driven tools. On the basis of the presented mathematical model, an algorithm was developed to generate a code that controls the machining of cylindrical gears with an involute profile of straight teeth, with the possibility of modifying the transition profile and the tooth root. The machining was experimental, and the gear was made of aluminium AlSi1MgMn using a solid carbide cylindrical cutter. In conclusion, the presented method was found to be very competitive with commercial methods and is able to provide very high quality gears. The accuracy of the machined profile form deviation in the entire processing did not exceed an average value of 10 μm; while assessing the tooth line, the basis average error was 5 μm. Finally, the gear was assessed as manufactured in accuracy class 6. This machining method guarantees very competitive machining cycle times, and thanks to the use of an uncomplicated CNC lathe and universal tools, it provides great flexibility, at the same time giving the possibility of machining gears with arbitrary profiles.

## 1. Introduction

Gear transmissions and their applications gained more and more importance at the turn of the 18th and 19th centuries. It was also during this period that more theoretical studies of tooth theory began to appear; in 1765, Leonard Euler published his work with a mathematical description of the meshing of two gears, treating the problem for a gear that moves uniformly without friction, as described in this study by Radzevich [1]. The presented assumptions could then be met only by gears with involute or epicycloid tooth contours. Euler, only in theory, presented the methods of constructing a tooth profile as the exact and approximate methods; however, his development was never used in the construction of the first machine for cutting teeth, which was pointed out by Litvin [2]. The constructors of the gears were then struggling with problems that are very current, but on a smaller scale: what is the most adequate profile of the mating teeth for a given situation, how to build a gear that can transfer a constant speed, and how to make the gear with teeth as accurately as possible for a given profile. Brown [3] developed an application patent method of gear machining and designed the tool, a disk cutter. The patented technology consisted of mapping the shape of the cutting edge of the tool to the outline of the machined tooth. The disadvantage of the method is the low level of accuracy depending on the shape of the tool contour, which is subject to constant wear during machining, and the lack of versatility of the method; each set of tools can be used to machine a gear with a constant module. The advantage of the method is that it can be used on simple, non-complicated machine tools. This machining method is still used today.

Along with the intensive development of CNC machine tools and cutting tools at the end of the 20th century, many innovative solutions in the field of gear machining have been developed. An American patent developed by Scherbarth [4] presents a method of machining gears on numerically controlled machine tools. The tool used in this process is a milling disk cutter with replaceable inserts. When milling a tooth profile, the milling axis is positioned on a plane perpendicular to the tooth flank to be machined, and the cutting inserts rotate in an angular range encompassing all the surfaces normal to the tooth profile. The tool is not geometrically related to the contour of the machined tooth. This processing method, known as invo-milling, is very efficient, but requires the use of a kinematically complex machine tool. In the development of his patent, Vogel [5] presented a method of machining gears using a conical tool with a hyperbolical profile, using numerically controlled machine tools simultaneously in five axes. The machining of the spaces between the teeth is carried out through the envelope of the successive positions of the cutter blades. According to this method, many geometrical variants of gears can be processed, including internal gears. The power-skiving method requires use of a special tool, geometrically adapted to the applied machining kinematics. The developed patent was an excellent basis for the further development of the method, as evidenced by subsequent studies by Harmut et al. [6] and Sture [7] covering issues with the development of the geometry of cutting inserts in tools used for machining. The new machining methods also had a great influence on the development of the geometrical aspects of gear design. Litvin et al. [8] developed a solution that improved the gear drive of the helical or spur types comprising a driving gear with a tooth surface that is uniquely double crowned in the profile and longitudinal directions and a driven gear provided with a conjugated tooth surface that can comprise a conventional involute type tooth surface or a uniquely modified tooth surface. Guttman et al. [9] presented a solution for elliptical modification of the tooth root; such solutions are now quite often used in gear constructions. The works of many other inventors included both issues related to the machining method and theoretical descriptions modifying the geometry of the tooth itself. Budzik et al. [10] presented a method of manufacturing gear by incremental processing and then by subtractive methods, giving the geometry of the gear its final shape. Due to the method used, the gear wheel in the invention has a reduced mass while maintaining a high stiffness. Buseler [11] also presented a method of constructing a gear with a reduced mass and increased stiffness; the proposed solution was also characterized by a very high susceptibility of the structure to vibration damping. Kostron et al. [12] presented a solution of a hybrid tooth profile in which the tooth head has an epicycloid shape and a hypocycloid root area. The epicycloid and the hypocycloid roll from the same point on the base circle, so the curves have one point in common. The rolling wheel is aligned with the pitch circle, so the tooth contact point is on the pitch circle. The possibility of modifying the tooth line with the use of classic machining methods was presented by Batsch [13], who proposed the use of the method of machining with a milling disk cutter. This modification is obtained by moving the wheel and the tool relative to the gear face width of the machined wheel. The method can be used in particular for the machining of the tooth line modified according to the Novikov method presented in a patent application [14]. An analytical solution for CNC milling in fivr axes with the use of a predefined set of milling tools was presented in the work of Pengbo et al. [15]. The proposed method ensures a very precise machining, thanks to the appropriate approximation between the input data of any surface form and its approximation of the envelope. The FEM analysis in the field of TCA in his work was presented by Shuting [16], focusing on the calculation of contact stresses and bending pairs of gears with machining errors. The results were compared with the plastic deformation of real gears. Increasingly, due to the rapid development of polymeric materials and composites, gears are made of non-metallic materials. This approach forces the gear designers to carry out strength and operational analyses of the gears. Landi et al. [17] and Chavadaki et al. [18] drew attention to the study of gears made of polymer materials, with particular emphasis on the possibility of optimizing the tooth root area, ensuring greater resistance to tooth bending. In his work, Gnatowski et al. [19] also drew attention to the possibility of using polymer materials as a constructional material for gears. The impact of thermomechanical properties of materials on the manufacturing process during the processing of gears with modification to the longitudinal tooth line was assessed.

The use of numerically controlled multi-axis machine tools in the production of gears gives manufacturers the possibility of a flexible approach in areas related to the shape and modification of the tooth profile, as evidenced by the publications cited earlier. The flexibility and versatility of production comes at the expense of production efficiency. Continuous operation of a CNC machine operating only in a narrow area of its technological capabilities has a large impact on its wear. Production errors in the topography of the tooth side surface resulting from machining and the tooling used in the process can be quite significant; therefore, the machined gear must be subjected to frequent checks on coordinate measuring machines using specialized software, which was pointed out in a study by Gosselin [20]. Continuous improvement of CNC machining methods allows for the efficient reduction of lead time in production, as described by Malek et al. [21]. Many methods of gear machining are a big challenge due to the production throughput; this problem was described by Kobialka [22]. The solution of adapting a conventional lathe with the use of special tooling for gear machining was presented in Gadakh [23]; such a solution while maintaining machining accuracy may ultimately guarantee a reduction in production costs. Kawasaki et al. [24] presents a method of regenerating large size bevel gears with the use of a CNC machining centre: after measuring on a coordinate measuring machine, the tooth surface was formalized, thus ensuring good working conditions for the gear after machining. The machining of large size gears on CNC machine tools is a very big challenge for designers and technologists. A very important role here is the possibility of simulating the machining process in order to avoid the possibility of a mistake, which was presented in an article by Yang et al. [25] as a method of solving the problems related to the machining of bevel gears with a spiral tooth line. As previously mentioned, the power-skiving method is one of the most effective methods of producing gears. The accuracy of machining in this method is dictated by the design considerations of the tool, and the design of the tool itself is complex and computationally demanding and requires special attention to the phenomenon of interference, which was indicated in a study by Chung-Yu [26]. The method of parametric programming of CNC machine tools and the use of this method for the machining of gears was presented in a work by Golebski [27]. This machining method has been practically implemented, and the machined gear wheel has the expected assumptions of machining accuracy. This work was the basis for the further development of work in the field of gear machining with the use of CNC machine tools. Golebski and Boral [28] presented the practical implementation of the multi-passing machining method on a CNC milling machine. The presented method of machining also included the possibility of producing a gear with longitudinal modification of the tooth line and profile. The idea of using geometrically simple tools in the machining of gears, and thus simple machines, is still a big challenge. On the basis of previous experience, this paper deals with the subject of experimental machining of gears on a CNC lathe with the use of driven tools while maintaining a high machining efficiency regime. The method indicated in the article also allows for easy modification of the tooth profile itself and, if necessary, the root area and head of the tooth.

## 2. Materials and Methods

The paper presents a case of machining an involute profile of spur gears as a two-parameter reeling of a tool along a given profile. The presented method is universal; any profile obtained computationally can be machined. The tool used is a geometrically simple cylindrical cutter, not related to the tooth profile being machined. The great advantage of this method of machining is its very high efficiency. The machining is carried out over the entirety of the working tooth width, and its efficiency largely depends on the machine tool and the type and diameter of the tool used. In the first stage of machining, the roughing allowance is removed, and the tool cuts in the radial direction of the gear, leaving the finishing allowance equidistant from the computational outline. The size of the finishing allowance should be the same along the entire contour of the tooth, so as to ensure that the angle of inclination of the tool in the material changes as little as possible during finishing.

### 2.1. Defining the Geometrical Parameters of the Tool Path

The basic parameters for determining the geometry of a cylindrical gear with straight teeth are:

*z*—number of teeth
*m*—module
*c*—top clearance
*y*—tooth height coefficient
*α*—pressure angle
*x*—coefficient of profile shift

**Figure 1:**
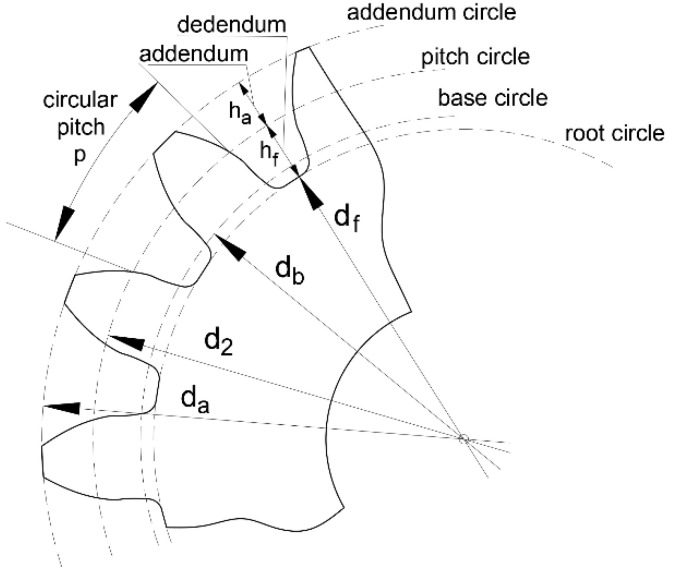
An involute gear and geometrical parameters.

The geometric parameters of the straight tooth involute gear (Figure 1) can be calculated from these equations:




c=0.1÷0.25


-top clearance coefficient

$l_{o}=c\times m$


-top clearance

$d_{2}=z\times m$



$r_{2}=\frac{d_{2}}{2}$

-pitch circle diameter

$h_{a}=y\times m$


-addendum

$h_{f}=y\times m$


-dedendum

p=π×m


-circular pitch

$d_{a}=d_{2}+2h_{a}$



$r_{a}=\frac{d_{a}}{2}$

-addendum circle diameter

$d_{f}=d_{2}-2\;h_{f}$



$r_{f}=\frac{d_{f}}{2}$

-root circle diameter

$d_{b}=d_{2\;}\cdot \cos\alpha_{0}$



$r_{b}=\frac{d_{b}}{2}$

-base circle diameter


Gears with teeth without correction were used for the analysis (*x = 0; k = 0*) where tooth height coefficient is (*y = 1*) and the top clearance coefficient is (*c = 0.20*).

The radius of the tool for machining the transition curve of the tooth side to the tooth area is:

(1) $$r_{t}=\frac{l_{0}}{1-\sin\propto_{0}}$$

The tooth profile was assumed as an involute profile delineated by a point of a straight line rolling along the base circle of the gear wheel. The angle between the radius of the involute’s initial point and the radius of the considered point of the involute is equal to the tangent of the outline angle of the involute point minus the outline angle of this point. This angle is the involute of the outline angle and is called involute function. The outline point on the pitch circle is equal to:

(2) $$\mathrm{inv}\propto_{0}={\mathrm{tg}\;\alpha }_{0}-\;{\underset{\alpha }{¯}}_{0}$$

where the ${\underset{\alpha }{¯}}_{0}$ angle in the arc is defined:

(3) $${\underset{\alpha }{¯}}_{0}\;=\;\frac{\alpha_{0}\times \;\pi }{180}$$

**Figure 2:**
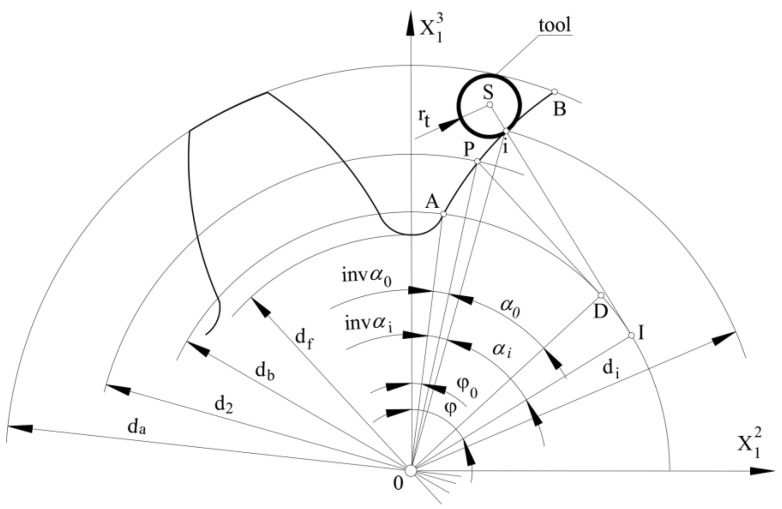
An involute outline of a machined tooth.

The coordinate system of the tooth profile is shown in Figure 2. The segment of the pitch circle arc from the ordinate axis to the point P is:

(4) $$\frac{\pi \;\times \;m}{4}=\frac{d_{2}}{2}\;\left(\varphi_{0\;}+{\mathrm{inv}\alpha }_{0}\right)$$

accordingly,

(5) $$\varphi_{0}=\frac{\pi \;\times \;m}{2\times {\;d}_{2}}-{\mathrm{inv}\alpha }_{0}$$

The angles of the contours for the beginning point *A* and the end point *B* of the tooth involute profile are equal:

(6) $$\alpha^{A}=0$$

(7) $$\alpha^{B}=\arccos\frac{d_{b}}{d_{a}}$$

that is, according to the adopted designation:

(8) $$\alpha^{P}=\alpha^{0}$$

To develop a machining program, it is necessary to save the tooth profile in the form of a discrete set of points for successive points of the profile with indices:

(9) i=1 .  .  .  n

the radii of successive outline points can be determined from the equation:

(10) $$\frac{d_{i}}{2}=\frac{d_{b}}{2}+\frac{d_{a}-{\;d}_{b}}{2\left(n-1\right)}\;\left(i-1\right)\;$$

On the other hand, the angles of the outline are analogous to the dependencies of (6) and (7)

(11) $$\alpha^{i}=\arccos\frac{d_{b}}{d_{i}}$$

The angle between the ordinate and the leading radius of the point of tangency normal to the outline at this point with the base circle is equal to:

(12) $$\varphi ={\;\varphi }_{0}+\alpha^{i}=\varphi_{0}+{\mathrm{tg}\alpha }^{i}$$

Ultimately, the coordinates of any point of the tooth profile for the right side of the tooth can be written in the coordinate systems as in Figure 2:

(13) $$X_{1}^{2R}=\frac{d_{b}}{2}\;\left(\sin\varphi -\varphi \cos\varphi \right)\;$$

(14) $$X_{1}^{3R}=\frac{d_{b}}{2}\left(\cos\varphi +\varphi \sin\varphi \right)$$

Thus, the position of the cutter axis (tool setting) for shaping any point of the involute tooth profile can be determined from the equation:

(15) $$X_{1}^{2\;\mathrm{Sr}}=\frac{d_{b}}{2}\sin\varphi -\left(\frac{d_{b}}{2}\varphi +r_{t}\right)\cos\varphi$$

(16) $$X_{1}^{3\;\mathrm{Sr}}=\frac{d_{b}}{2}\cos\varphi +\left(\frac{d_{b}}{2}\varphi +r_{t}\right)\sin\varphi$$

However, for the outline on the left side (right side of the tooth) it will be equal to:

(17) $$x_{1}^{2^{\left(l\right)}}=-{\;x}_{1}^{2^{\left(r\right)}}$$

(18) $$x_{1}^{3^{\left(l\right)}}={\;x}_{1}^{3^{\left(r\right)}}$$

(19) $$x_{1}^{2^{\left(\mathrm{Sl}\right)}}=-{\;x}_{1}^{2^{\left(\mathrm{Sr}\right)}}$$

(20) $$x_{1}^{3^{\left(\mathrm{Sl}\right)}}=-{\;x}_{1}^{3^{\left(\mathrm{Sr}\right)}}$$

In the developed tooth profile processing technology, it is important to be able to easily modify the tooth profile. Very often, the modification concerns only the contour area that is not involved in the meshing, i.e., the root and the head of the tooth. The transition curve of the tooth profile side to the bottom land should be a tangent to the involute profile at the profile origin. Two machining cases can be defined to process a fragment of the tooth profile below the base circle. The first case concerns the machining that takes place in one cycle when cutting the main contour in the finishing cycle, assuming that it will be a milling cutter with a radius equal to the radius of the tooth bottom land rounding, or the radii of the rounding of the tooth sides at the transition to the bottom. In this case, the tool inclination angle in the material is larger, and it may cause a deterioration of the structure of the machined surface. In the second case, we can shape the transition contour with a milling cutter with a radius smaller than the radii of the rounding of the side contours at the bottom land. This method requires a machining strategy with one or two tools. The tools must maintain a smooth transition between the outline and the transition curve towards the tooth bottom. In the solution adopted for this research, one milling cutter with a diameter smaller than the width of the bottom of the tooth profiles was used, thanks to which, even taking into account the allowances for finishing on both sides of the tooth, the tool could work in the areas of the inclination angle, ensuring stable operation without vibrations. The adopted machining strategy was also determined by a higher efficiency (machining the entire gear with one tool) and the possibility of performing a qualitative assessment of machining. The method assumes that in the process of machining the transition curve, as well as in the entire process of machining the tooth profile, the gear wheel performs a relative rotational movement C around the Z axis, while the shaped bottom land takes a symmetrical position with respect to the adopted coordinate system for the needs of the profile calculations.

#### Tooth Profile Modification

**Figure 3:**
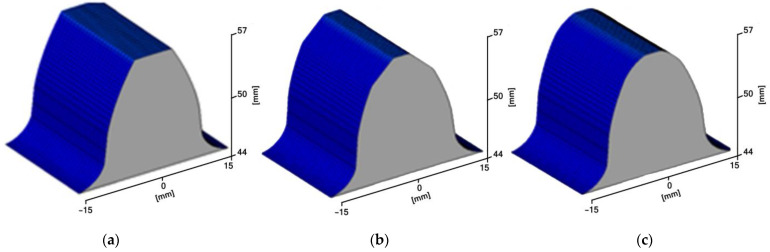
Tooth profiles. (**a**) involute without modification, (**b**) involute with modification along an arc of the head of the tooth, (**c**) involute with modification along a straight line of the head of the tooth.

In the tested method, we can very easily apply a modification of the outline, which may consist of replacing the tooth forming profile at the considered point of the outline with an arc of a circle or a straight line. Such a solution can be used to improve the quality of gear operation, and it can be used for gears with a limited number of teeth in order to avoid the interference phenomenon. Figure 3a shows an analytically obtained involute tooth profile—without modification of the root and tooth head; such a case was processed on the machine and further analysed. Figure 3b,c show the modified contours; they can be machined according to the same machining strategy, as the size of the modification does not affect the nature of the process and is only determined by the diameter of the milling cutter used.

The tool used is a cylindrical cutter that is not geometrically related to the machined tooth profile (the tool and the machined gear are not mutually enveloped). It is a classic case of a two-parameter circumference (the form of the machined surface does not depend on the tool geometry). The developed calculation algorithm is universal and can be used to prepare the technology for the machining of any tooth, both external and internal, on a numerically controlled lathe with the use of driven tools.

### 2.2. Machining Process

**Figure 4:**
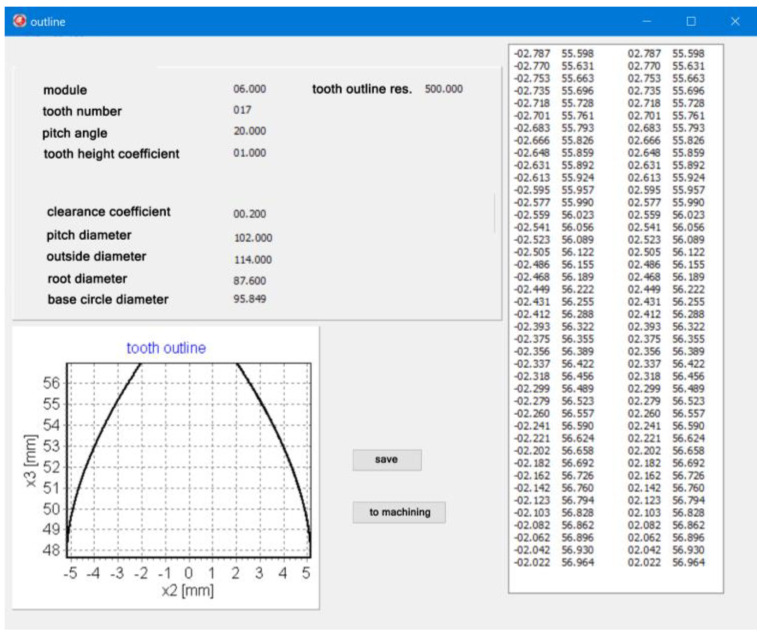
A tooth outline and tool path generating software.

Based on the analysis of the discrete record of coordinates, an application supporting the calculation of the tooth profile was developed. The software consists of two modules. In the first module, after reading the basic parameters of the toothed wheel, *z* = 17, *m* = 6, *α* = 20°, *c* = 0.2, *x* = 0, *y* = 1, a discrete record of the outline coordinates is generated (Figure 4). These are the coordinates that define one plane of the tooth profile. By extending it in the direction of the width of the rim of the wheel, it is possible to generate a solid model of the machined tooth to verify the correctness of the calculations (Figure 3a). The tool path can be saved in a separate text file, in which the tool diameter has been taken into account.

**Figure 5:**
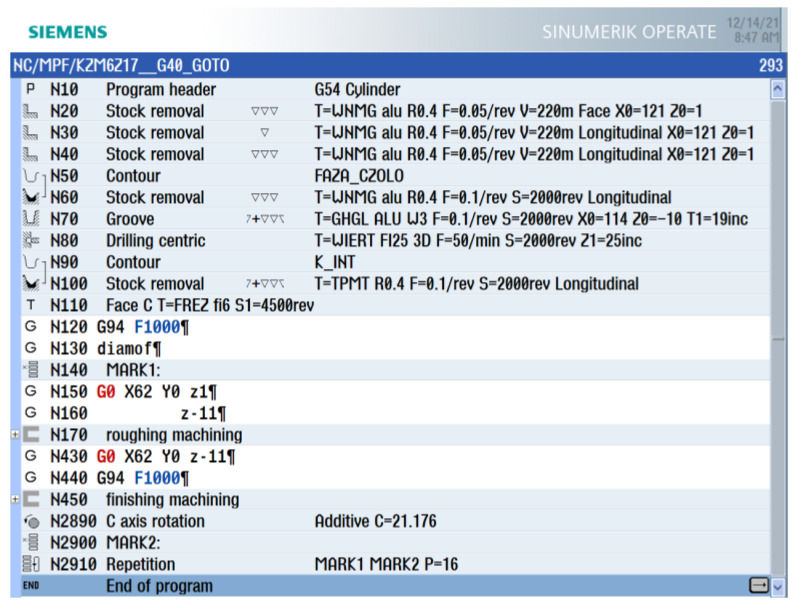
The machining program integrated with the Sinumerik 840 Dsl control system.

The obtained tool path coordinates take into account the resolution of the tooth profile calculations, which should be adjusted depending on the size and module of the machined gear. The entire generated program includes machine control functions in accordance with the ISO code. It is so universal that it can be adapted to any machine tool that can be programmed according to standardized functions. The generated toolpath is saved in a text file and can be merged with the main program code of the indicated control system. When adapting the tool path obtained from the calculations, it is necessary that the contours generated in the program can be recreated without the program declaration of tool radius compensation. The tool radius compensation is included in the tool path calculation step. Figure 5 shows the machining program integrated for the Sinumerik 840 Dsl control system.

Program blocks 20–100 include machining for blank parts of the gear, including the centering bore, while lines 140–2900 contain the program code for machining one space between the sides of the teeth—the left side of the tooth profile (right) and the right side of the tooth profile (left). Block 2890 includes a split command for machining the next gear segment. Subsequently, the program between markers MARK1 and MARK2 is repeated 16 times. The gear with an involute profile with the geometrical parameters indicated in Table 1 was machined. The width of the gear rim of has been limited to 10 mm so that the tool is not significantly loaded during the machining process.

**Figure 6:**
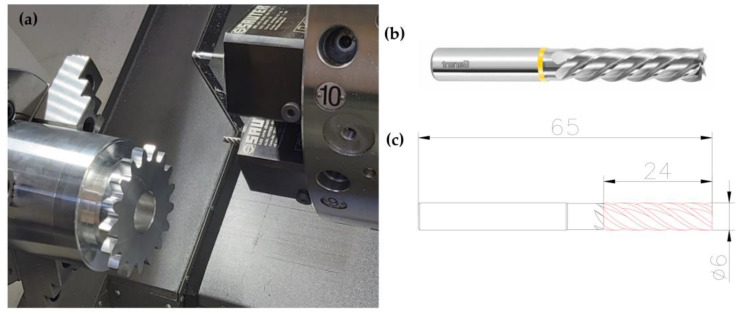
(**a**) The machined gear mounted in a three-jaws chuck. (**b**) The solid carbide cylindrical milling cutter used in processing, (**c**) The dimensions of the milling cutter [29].

A numerically controlled lathe from DMG MORI, model CLX350V4, equipped with a Sinumerik 840D sl control system, was used for machining. The machine is also equipped with the ShopTurn programming overlay, allowing for conversational dialogue programming. The blank was made of a uniform AlSi1MgMn aluminium material, mounted in the three-jaw self-centering chuck of the lathe (Figure 6a). The Mahr MarCator 1075R (Mahr GmbH, Göttingen, Germany) measuring sensor with a measuring resolution of 5μ and a reading resolution of 1μ was used to check the correct mounting—parallelism of the face of the gear in relation to the lathe spindle. By adjusting the clamping force of the jaws, it was possible to obtain a parallelism deviation in the X axis of 6μ. For machining, a cylindrical cutter—ϕ6 mm, VHM, with high accuracy, with polished chip flutes for better chip evacuation—was used (Figure 6b,c). A cylindrical milling cutter with 5 blades, a blade diameter tolerance not exceeding 4μm and the maximum allowable milling width of 0.2 mm for a Ø6 tool was used. During machining, the chips were discharged with compressed air. The maximum rotation speed of the tool is 8000 rpm and the feed rate is 0.385 mm/rev. A tool shank according to DIN 6535 HA in h6 tolerance was used. A driven tool holder with a straight geometry (parallel to the spindle) was used, SAUTER VDI30 (Figure 6), with a mounting type ER25 and maintained a rotational accuracy of ≤2 µm.

**Figure 7:**
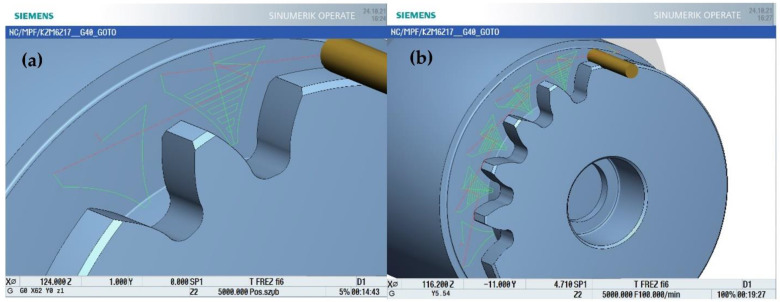
The adopted machining strategy. (**a**) the roughing and finishing tool path, (**b**) the machining strategy.

The developed machining program included roughing—a plunge division of the space between left and right tooth profile at the level of 10 layers (Figure 7a)—after which an equidistant allowance for finishing of 0.15 mm was left on the profiles. The allowance was removed in one finishing pass of the tool according to the calculated trajectory movement (Figure 7b).

The involute line was built of straight sections, and its resolution depends mainly on the processing capabilities of the machine control system itself. In the examined case, the calculations assumed the resolution of the involute outline in the form of 500 straight line segments for one side of the tooth profile. Therefore, it is not difficult to calculate that the complete machining of one space between tooth profiles for the tested method is about 1050 program lines. During the test work of the machining, an important thing was noticed that influenced the efficiency of the program implementation. To ensure efficient operation of the machine control system, the main machining program should cooperate with roughing and finishing subroutines that were sequentially loaded into the main program and executed. This solution significantly shortened the time of the graphical presentation of the program on the machine’s desktop. One tool was used for roughing and finishing in the entire process. This assumption was to allow the assessment of the effect of its wear on the condition of the machined surfaces and the accuracy of the entire gear.

## 3. Results

**Figure 8:**
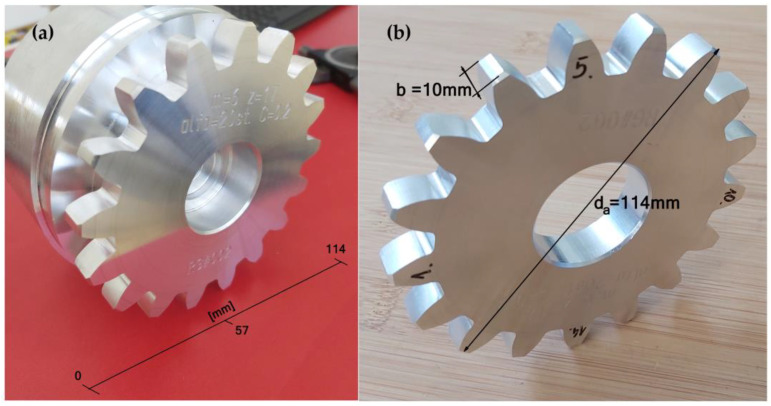
The machined gear. (**a**) the machined gear, (**b**) the cut off gear rim with indicated tooth samples machined with different feed rates.

The machined gear (Figure 8a) was tested for the geometry parameters on a coordinate measuring machine, then four sections of the gear were subjected to surface tests after machining, and four teeth were cut off (Figure 8b). Such actions ensured free access to the tested surfaces during the measurement.

### 3.1. Measurement of Geometrical Compliance of a Gear with the Use of Software Module ZEISS Gear Pro Involute

Achieving small tolerances of the mating teeth is the most important factor responsible for the proper operation of the gearbox. More and more demands are made for modern numerically controlled machine tools in terms of accuracy and repeatability of machining. Thanks to this, the expectations regarding the accuracy of gear manufacturing and their testing are increasing. According to ISO 1328 [30]: cylindrical gears and the ISO system of flank tolerance classification, the parameters of gear wheel performance were measured and classified using the GEAR PRO module of the ZEISS CALYPSO software (Version ZEISS CALYPSO 2020, Jena, Germany). The following parameters of the gear wheel were determined during the measurements:

*Fα*—profile deviation, the total overlay of the profile form deviation and the profile slope deviation;
*f_fα_*—form deviation of the profile without consideration of the slope deviation;
*f_Hα_*—profile slope deviation of the profile without consideration of the form deviation;
*Fβ*—tooth line (lead) deviation, total overlay of the lead form deviation and the lead slope deviation;
*f_fβ_*—form deviation of the lead without consideration of the slope deviation;
*f_Hβ_*—lead slope deviation of the outline without consideration of the form deviation.

In addition, the parameters of the pitch of the gear wheel were measured:

*Fp*—total cumulative pitch deviation, the range of the positional deviation of all the right (left) flanks to the nominal position, with the flanks being analysed independently;
*fp*—single pitch deviation, the maximum unsigned positional deviation of all the right (left) flanks to the preceding right (left) flank;
*fu*—adjacent pitch difference, the maximum unsigned difference of all the individual single pitch deviations of all the right (left) flanks.

**Figure 9:**
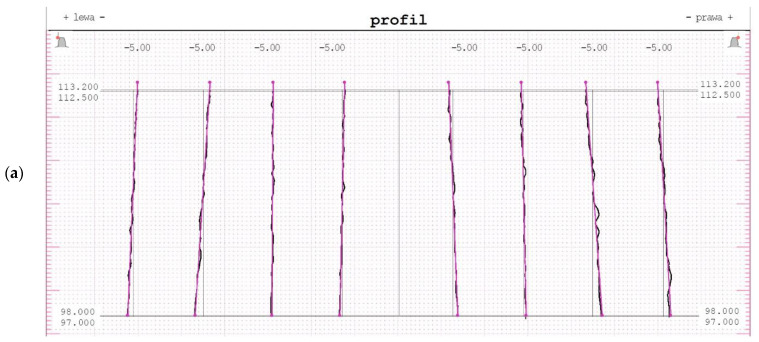

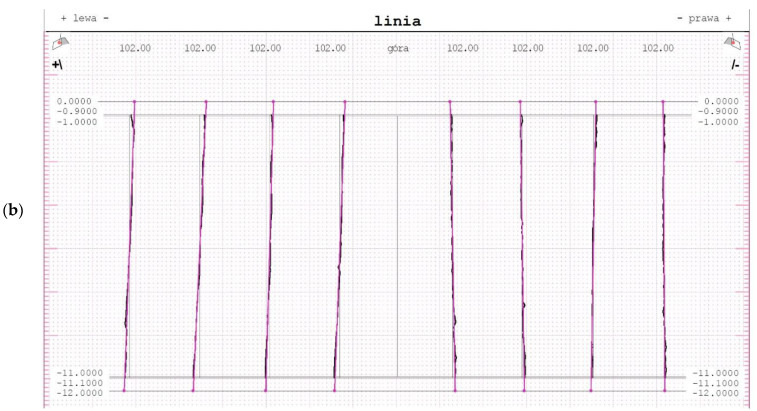
A graphical presentation of the geometrical gear measurement results. (**a**) the profile measurement results, (**b**) the tooth line measurement results.

Table 2 shows the tolerance fields of the indicated measurements on the basis of the averaged results of the measurements of the gear. The graphical presentation of measurement results are shown in Figure 9. Measurements were made on a coordinate measuring machine using the GEAR PRO Involute module of the ZEISS CALYPSO software. The software enables the inspection of spur gears, bevel gears and worms. At the beginning, it should generate a CAD model of the gearing from the geometry definition. The interface allows the operator to track their input values. Measurement can be started immediately after the geometry has been defined according to the programme’s measurement plan default settings. Several measuring runs can be defined per measurement plan, and the results can also be submitted directly for statistical evaluation. The measurement can be performed on a standard coordinate measuring machine, as the software enables measurements with or without a rotary table.

According to the ISO standard [31], on the basis of the obtained measurement results presented in Table 2, the gear manufacturing accuracy class was determined. The obtained profile deviations classify the gear to the manufacturing class 5 (Figure 9a). When assessing the tooth line on the basis of the obtained parameter results, the gear was assessed as manufactured in accuracy class 6 (Figure 9b). It should be mentioned that the size of the obtained deviations was mostly influenced by the method of processing, with particular attention to the fixing of the blank parts in the lathe chunk. The accuracy of the profile of the machined gear was very high, where the total overlay of profile form deviation in the entire machining did not exceed 10 μm. The tooth lines are also characterized by a repeatable and correct shape, and the average total overlay of lead form deviation was only 5 μm. The parameter of total cumulative pitch deviation was determined for the whole gear at the level of 31 μm, with a maximum unsigned positional deviation of 11 μm.

### 3.2. Measurement of the Structure of the Tooth Surfaces

**Figure 10:**
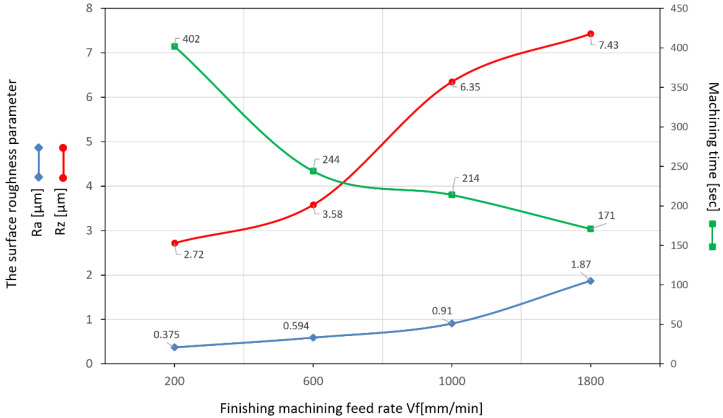
The measured roughness profile Ra and Rz parameters, measured longitudinally to the tooth profile. The results of machining time as a function of feed rate.

In the next stage of the analysis, the gear was assessed on a Taylor Hobson Talysurf 120 (Taylor Hobson, New Star Road, Leicester, UK, 2012) laboratory contact profile graphometer. A measuring blade with a 2 µm tip was used for the measurement. The roughness of the machined tooth profiles was measured in the direction of the profile in the half of the gear rim width in four sections, machined in various ranges of feed speeds (Figure 10). Ra is the arithmetic mean roughness value from the amounts of all profile values. Ra does not differentiate between peaks and valleys, and therefore has a relatively weak information character, while Rz presents the maximum height of profile as the average value of the five Rz values. The Ra parameter reacts poorly and has local changes in the surface structure, so its value often does not give a clear picture of the surface condition. For this reason, the roughness parameter Rz was additionally selected to describe the surface condition. The machining times were simulated depending on the feed speed of the tool. In principle, roughing machining was carried out with the same feed value of 1800 mm/min. This is the nominal value for the tool used, taking into account the rotational speed of 4500 rev/min, the maximum for the driven holder used. It should be noted that the length of the tool path in finishing in relation to roughing is 40/60.

**Figure 11:**
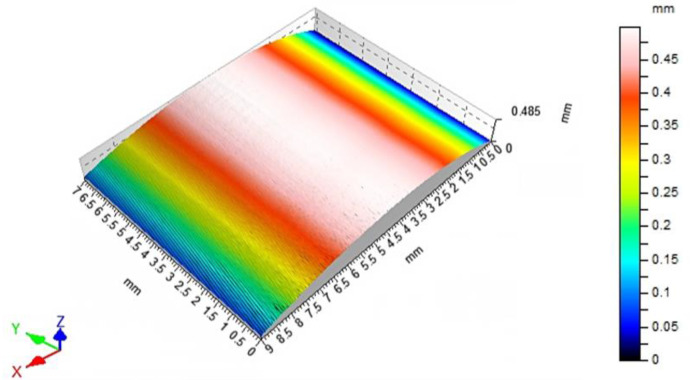
A stereometric distribution of the machined tooth—sample 4, machined with a feed speed of V_f_ = 1800 mm/min.

**Figure 12:**
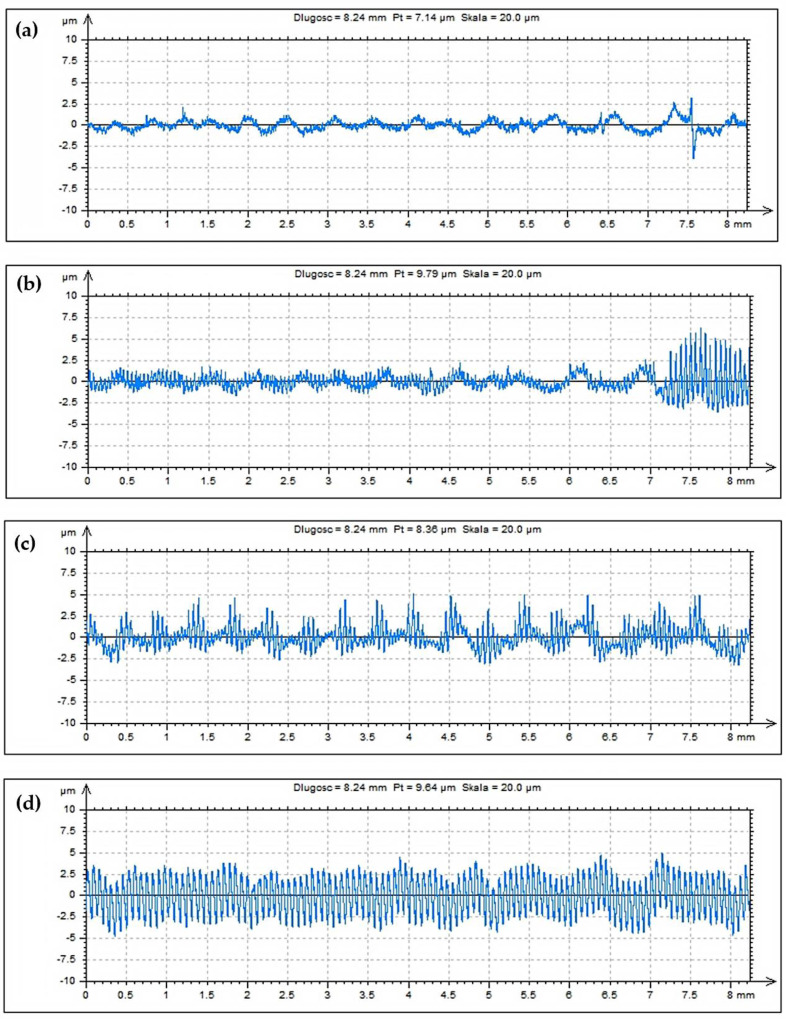
The roughness parameter profile Ra measured in the direction of the tooth profile. (**a**) feed rate V_f_ = 200 mm/min, (**b**) feed rate V_f_ = 600 mm/min, (**c**) feed rate V_f_ = 1000 mm/min, (**d**) feed rate V_f_ = 1800 mm/min.

A number of measurements were made, including the surface roughness parameters Ra and Rz, in order to better represent local changes in the surface structure **(**Figure 10). The machining process was carried out within the normative range of the cutting parameters. It can be noticed here that the roughness parameter Ra, in the entire extent of its changes, does not present above-normative deviations. Roughness changes in the range of feed speeds from 200 to 1000 mm/min are slight and acceptable for maintaining a high-quality machining process. The results of the measurement of the roughness parameter Rz are also satisfactory; it is very important for the assessment of sudden increase of local changes in the roughness profiles caused by phenomena in the machining process. The increase in the Rz parameter value is correct in relation to the obtained Ra parameter results. The stereometric distribution of the machined tooth area was also determined (Figure 11). The microgeometric structure of the top layer was also assessed, and the measurement was made in the middle section of the tooth perpendicular to the processing direction. Figure 12 shows the original structure of the surface profile; the shape of the involute tooth profile was filtered.

**Figure 13:**
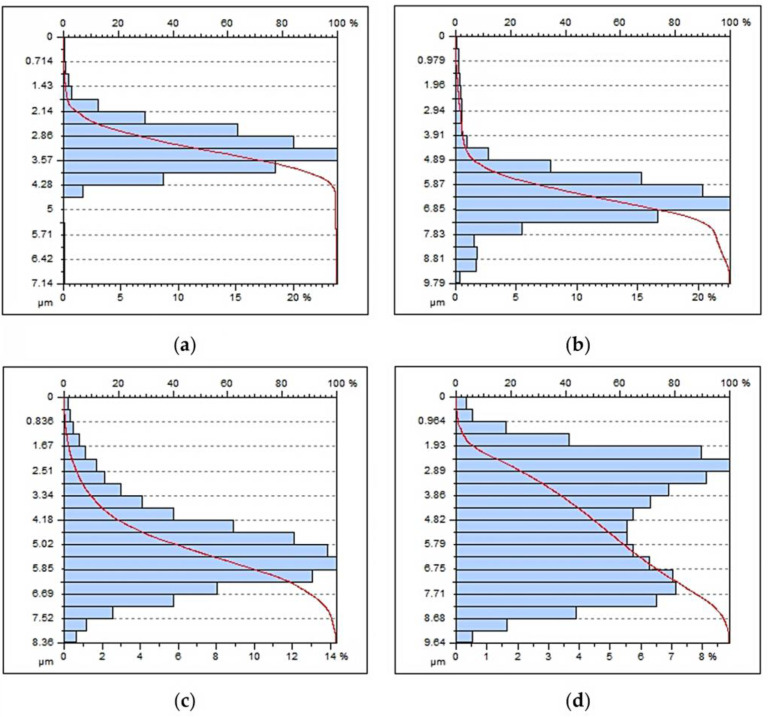
The material ratio as a function of the height, Abott-Fireston curve. (**a**) feed rate V_f_ = 200 mm/min, (**b**) feed rate V_f_ = 600 mm/min, (**c**) feed rate V_f_ = 1000 mm/min, (**d**) feed rate V_f_ = 1800 mm/min.

The main profile parameters are defined in the ISO 4287 standard [31]. Measured profile is a series of height values as a function of the lateral position. Figure 12 shows the roughness profile from the fillet area to the tooth head for the four analysed machining variants. The roughness profile distribution is constant in all cases, only in the case of tooth fillet area (Figure 12b), there was a local, abnormal deterioration of the surface condition. This may result from the enlargement of the area of the tool contact zone, which was often accompanied by chatters during machining. The heights are references by convention from the mean line, which is calculated as the mean value of all heights. Figure 13 shows the functional parameters of the surface Rmr as the material ratio at a given depth. This parameter gives the percentage of material cut at a given depth from the top of the profile. The reference may also be taken from the centre line or another reference height.

## 4. Discussion

The machining of gears on CNC machines places very high demands. The industrial method of machining of gears, invo-milling [4], allows for the manufacture of a gear with accuracy class 4 without the use of finishing technology with grinding. From the point of view of energy consumption of the entire process, this is a very big advantage. Unfortunately, in this method, machining is carried out on an expensive and kinematically complex machine tool. For the experimental method described in this paper, it was important to check the geometric parameters of the machined gear as well as the quality parameters of the surface. When evaluating the operational properties of the gear, a very important research result was the evaluation of the gear parameters using the specialized Gear Pro Involute software. The above results may also prove the high class of the machine tool used for machining. The used CLX350V4 lathe is an underused machine with a total time work of 120 h of the spindle. In addition, the machine is equipped with an absolute position measuring system in the X and Y axes, which significantly ensures the repeatability of positioning. The gear machining was carried out in the simultaneous cycles of the X, Y, C axis movement. Therefore, it was also very important to determine the parameters related to division errors during the spindle’s C axis movement. The obtained results with the experimental nature of the machining can be regarded as very satisfactory. In the process of machining gears, with particular emphasis on CNC machines, machining efficiency also plays a very important role. For this reason, it was also important to determine the machining parameters at which the quality parameters of the surface would be maintained. The obvious conclusion is that the quality parameters of the surface will deteriorate with the increase of the feed speed; the aim of the study, however, was to determine precisely to what extent. Figure 11 shows the complete stereometric image of the machined surface. The surface did not deteriorate excessively, maintaining the uniformity of the structure over the entire extent of the area of the useful side of the tooth. The root area shows no changes related to the increased inclination of the tool during machining. This area of the tooth does not participate in the cooperation during meshing, therefore it is justified in this case to make a geometric modification of the root area in order to improve the strength properties of the tooth fillet while reducing the tool inclination during machining. Figure 13a–d shows the surface function parameter as a percentage of material cut at a given depth. The bearing area curve (BAC) allows for the description of the diversity of the properties of the tested profile that changes with its depth. The BAC, or the Abott-Fireston curve, determines the percentage of the material cut in relation to the material being covered for a given depth (vertical axis of the graph). The surface load coefficient as a percentage is represented on the horizontal axis. Based on the three BAC parameters—the reduced height of the rises, the average height of the rises above the core of the roughness profile, the depth of the roughness core, the reduced depth of the pits and the average depth of the pits under the roughness profile core—we can forecast the characteristics of the tooth surfaces operation process. For samples (a), (b) and (c), the size of the material core is the smallest with an increased value of profile heights. Such an arrangement causes the contact area with another surface to be small while increasing the surface unit pressure. The sample (d) processed with the fastest feed parameters is characterized by a very large depth of the roughness core, while the large parameter of the reduced depth of the recess improves the ability of the surface to hold the lubricating medium. From the point of view of maintaining better operational capabilities of the transmission, machining the tooth profiles with higher speeds is justified in this case.

## 5. Conclusions

In summary, it can be stated that the experimental method of gear machining on a numerically controlled lathe with the use of driven tools presented in this paper has a number of advantages:

- Machining with a tool that is not geometrically related to the contour being machined, in our case, as a two-parameter circumference, is an excellent alternative to complicated and expensive hobbing methods.
- This method allows the use of any modifications of the machined tooth profile; moreover, the processing of profiles other than involute with non-standard modules is not a problem in this case.

The main problem of the application and further development of non-commercial methods of gear machining is their insufficient efficiency combined with low quality. In the analysed case, the conducted research proves that it does not have to be this way:

- This method guarantees very efficient machining and at the same time guarantees the quality of the process. The graph in Figure 11, on the basis of the performed machining simulations, presents the time dependence in the function of feed changes. As can be seen, the feed rate is the biggest factor influencing the efficiency of the entire gear machining process.
- The obtained machining time of less than three minutes for the cutting variant at a feed rate of 1800 mm/min is an excellent result, and the analysis of the quality of the machined surface in this case is also very good.
- The analysed machining process has significant reserves in terms of productivity; the driven tool holder with a maximum rotational speed of 4500 rpm significantly limited the feed rate used in machining.

The quality of the gear is determined by the lowest manufacturing accuracy of one of its components. The tested gear wheel, in terms of geometric and surface quality, obtained satisfactory results:

- The accuracy of the machined profile form deviation in the entire processing did not exceed average value of 10 μm, while assessing the tooth line, the basis average error was 5 μm.
- The measurement of the stereometry of the surface layer showed an even distribution of its structure. The roughness and material ratio indicators showed correct differentiation in relation to the applied changes in machining parameters.

The presented work proved that it is possible to machine gears on a basic CNC lathe, and the obtained results of geometric and surface accuracy, while maintaining technological discipline, may be fully satisfactory. This machining method can be used not only for machining external gearing, but also for the more technologically demanding processing of internal gears. It can also allow the production of gears with any modification to the tooth profile.

The method of gear machining described in the work has been submitted to the Polish Patent Office as a patent claim under the number P.439202.

## Figures and Tables

**Table 1:** The machined gear parameters.

Module [mm]	Number of Teeth	Pitch Angle [deg]	Tooth Height Coefficient	Clearance Coefficient [mm]	Face Width [mm]
6	17	20	1.0	0.20	10

**Table 2:** The measurement results of the tolerance fields.

Gear Parameter [mm]	Avg. Fα [μm]	Avg. ffα [μm]	Avg. fHα [μm]	Avg. Fβ [μm]	Avg. ffβ [μm]	Avg. fHβ [μm]	fp [μm]	Fp [μm]	fu [μm]
Measurement results	10	11	6	5	7	1	11	31	15

## Data Availability

Data are contained within the article.

## Abbreviations

*α*	pressure angle
$\alpha_{0}$	angle between the radius of the involute’s initial point and the radius of the considered point of the involute
${\underset{\alpha }{¯}}_{0}$	arc angle in involute function
φ	angle between the ordinate and the leading radius of the point of tangency normal to the outline
$\varphi_{0\;}$	angle from the ordinate axis to the point P on pitch circle
$\alpha^{A}$	angle of the contour for the beginning point A of the tooth involute profile
$\alpha^{B}$	angle of the contours for the end point B of the tooth involute profile
$\alpha^{P}$	angle of the contours for the point P on pitch circle
$\alpha^{i}$	angle of the contours for subsequent points

## References

[1] Radzevich P.S. (2012). Theory of Gearing: Kinematics, Geometry, and Synthesis.

[2] Litvin F.L. (1997). Development of Gear Technology and Theory of Gearing.

[3] Brown J.R. (1864). Improved Cutter for Cutting Gear-Wheels. U.S. Patent.

[4] Scherbarth S. (2016). Tooth Milling Cutter and Method for Milling the Teeth of Toothed Gear Elements. U.S. Patent.

[5] Vogel O., Nagele J. (2014). Power Skiving Tool for Power Skiving Gear Teeth on a Crown Wheel Workpiece. U.S. Patent.

[6] Harmut M., Vogel O. (2011). Semi-Completing Gear Skiving Process and Device with Corresponding Skiving Tool for Carrying out a Semi-Completing Skiving Process. EP Patent.

[7] Sture S. (2014). Cutting Insert and Power Skiving Tool. EP Patent.

[8] Litvin F.L., Feng P.H., Lagutin S.A., Townsend D.P., Sep T.M. (2001). Helical and Spur Gear Drive with Double Crowned Pinion Tooth Surfaces and Conjugated Gear Tooth Surfaces. U.S. Patent.

[9] Gutmann P., Hong L.J. (2009). Toothed Gear Manufactured by Involute Envelope Method.

[10] Budzik G., Dziubek T., Sobolewski B., Przeszłowski Ł. (2018). Toothed Gear, Manufacturing Method. PL Patent.

[11] Bauseler S. (2001). Toothed Gear. PL Patent.

[12] Kostron A., Karowiec K., Skrzypiec A., Serwotka R., Sedlaczek J. (1996). Toothed Gear. PL Patent.

[13] Batsch M. (2015). Modification Method of Tooth Line of the Gear. PL Patent.

[14] Boiko S.L., Korotkin V.I., Veretennikov V.Y., Roslivker E.G., Fedyakin R.V., Chesnkov V.A., Yakovlev A.S., Kharitonov J.D., Fei V.M., Galichenko E.N. (1991). Novikov Gearing. U.S. Patent.

[15] Pengbo B., Haizea G., Amaia C., de Lacalle L.N., Barton M. (2020). 5-axis double-flank CNC machining of spiral bevel gears via custom-shaped milling tools—Part I: Modeling and simulation. Prec. Eng..

[16] Shuting L. (2007). Finite element analyses for contact strength and bending strength of a pair of spur gears with machining errors, assembly errors and tooth modifications. Mech. Mach. Theory.

[17] Landi L., Srecconi A., Morettini G., Cianetti F. (2019). Analytical procedure for the optimization of plastic gear tooth root. Mech. Mach. Theory.

[18] Chavadaki S., Kumar K.C.N., Rajesh M.N. (2021). Finite element analysis of spur gear to find out the optimum root radius. Mater. Today Proc..

[19] Gnatowski A., Gołębski R., Sikora P. (2021). Analysis of the Impact of Changes in Thermomechanical Properties of Polymer Materials on the Machining Process of Gears. Polymers.

[20] Gosselin C. (2018). Multi Axis CnC Manufacturing of Straight and Spiral Bevel Gears. Advanced Gear Engineering.

[21] Malek O., Mielnik K., Martens K., Jacobs T., Bouquet J., Auwers W., Ten Haaf P., Lauwers B. (2016). Lead Time Reduction by High Precision 5-axis Milling of a Prototype Gear. Procedia CIRP.

[22] Kobialka C. (2010). Complete Machining of Gear Blank and Gear Teeth.

[23] Gadakh R.S., Londhe P.G., Shaikh B.A., Shaikh F.S. (2016). Gear Manufacturing by using conventional lathe machine. Int. J. Res. Eng. Technol..

[24] Kawasaki K., Tsuji I., Gunbara H., Houjoh H. (2015). Method for remanufacturing large-sized skew gears using CNC machining center. Mech. Mach. Theory.

[25] Yang L., Huang G. (2011). The OPC technology research about spiral bevel gear machine tools for machining simulation problems. Proc. Eng..

[26] Chung-Yu T. (2021). Power-skiving tool design method for interference-free involute internal gear cutting. Mech. Mach. Theory.

[27] Gołębski R. Parametric programming of CNC machine tools. Proceedings of the 4th International Conference on Computing and Solutions in Manufacturing Engineering 2016, Transilvania University of Brașov.

[28] Gołębski R., Boral P. (2021). Study of machining of gears with regular and modified outline using CNC machine tools. Arch. Mater..

[29] Perschmann Tools Guide Catalogue. https://www.hoffmann-group.com/GB/en/houk/Mono-machining/Solid-carbide-milling-cutters/GARANT-Master-Alu-solid-carbide-milling-cutter%2C-HPC-HPC-uncoated/p/203208-6.

[30] (2015). ISO 1328-1:2015.

[31] (1999). ISO 4287:1997.

